# Evolving treatments and outcomes in HER2-Positive metastatic breast cancer: Data from the GIM14/BIOMETA study

**DOI:** 10.1016/j.breast.2023.103583

**Published:** 2023-09-25

**Authors:** Massimo Di Maio, Claudia Bighin, Francesco Schettini, Tommaso Ruelle, Laura Marandino, Alessandra Fabi, Carmine De Angelis, Mario Giuliano, Pietro De Placido, Michelino De Laurentiis, Ferdinando Riccardi, Caterina Picotto, Fabio Puglisi, Lucia Del Mastro, Grazia Arpino, the Gruppo Italiano Mammella (GIM) Study Group

**Affiliations:** a Department of Oncology, University of Turin, Ordine Mauriziano Hospital, Turin, Italy; b Department of Medical Oncology, U.O. Oncologia Medica 2, Ospedale Policlinico San Martino, Genoa, Italy; c Department of Medical Oncology, Hospital Clinic of Barcelona, Barcelona, Spain; d Translational Genomics and Targeted Therapies in Solid Tumors, August Pi i Sunyer Biomedical Research Institute (IDIBAPS), Barcelona, Spain; e Facultat de Medicina i Ciències de la Salut, Universitat de Barcelona, Barcelona, Spain; f Department of Internal Medicine and Medical Specialties (DiMI), School of Medicine, University of Genova, Genova, Italy; g Department of Medical Oncology, IRCCS Ospedale San Raffaele, Milan, Italy; h Precision Medicine in Senology Unit, Fondazione Policlinico Universitario A. Gemelli IRCCS, Roma, Italy; i Department of Clinical Medicine and Surgery, Oncology Division, University of Naples Federico II, Naples, Italy; j Department of Breast and Thoracic Oncology, Istituto Nazionale Tumori IRCCS Fondazione Pascale, Naples, Italy; k Oncology Unit, Antonio Cardarelli Hospital, Naples, Italy; l Department of Medical Oncology, Unit of Medical Oncology and Cancer Prevention, Centro di Riferimento Oncologico di Aviano (CRO) IRCCS, Aviano, Italy; m Department of Medicine (DAME), University of Udine, Udine, Italy

**Keywords:** Breast cancer, HER2-Positive, Metastasis, First line of therapy, Trastuzumab

## Abstract

**Background:**

Treatment for HER2-positive (+) metastatic breast cancer has improved in the last decade. We analyzed treatment changes over time and their impact on patients outcomes in a real-world dataset.

**Methods:**

Data from 637 HER2+ patients with metastatic breast cancer enrolled in the multicenter Italian GIM14/BIOMETA study were retrieved. Progression-free survival (PFS) over time was evaluated according to the type of anti-HER2 therapy, disease onset (de novo vs. relapsing), metastatic site, and year of treatment (2000–2013 vs. 2014–2020).

**Results:**

Median follow-up was 64.4 months. Overall, for first-line therapies, mPFS was 16.5 vs 19.5 months for patients treated in 2000–2013 vs 2014–2020 (HR: 0.78, 95% CI:0.65–0.94, *P* = 0.008). mPFS improved over time in all patients except for those with brain metastasis. Interestingly mPFS was 17.4 vs13.4 months (HR, 1.49; 95% CI, 1.13–1.98, *P* = 0.005) in 2000–2013 and 24.4 vs 20.9 months (HR 1.04; 95% CI 0.78–1.40 p = 0.77) in 2014–2020 in pts without vs with liver metastases. For second line therapies, the overall median PFS was 9.6 months (95% CI, 8.31–10.97) and did not change over time.

**Conclusion:**

Median first-line PFS improved since 2014, mainly due to the introduction of pertuzumab. The outcome of patients with liver metastases appears to have improved in recent years. Patients with brain metastases had the worst PFS, which also did not improve over time.

## Highlights

• Overall, from 2000 to 2020, mPFS of patients treated with 1st-line with anti-HER2 agents improved over time.
• With the introduction of pertuzumab in 2014, mPFS significantly improved, especially in patients with liver metastases.
• Brain metastasis were associated with a significantly worse PFS regardless the year of treatment.
• For patients with brain metastases, novel, more active, therapeutic agents should be strongly advocated to improve outcome.

## 1 Introduction

Survival rates of patients diagnosed with human epidermal growth factor receptor 2 positive (HER2+) metastatic breast cancer (MBC) have substantially improved over the last two decades [[1], [2], [3]] thanks to the implementation in routine clinical practice of anti HER2 therapies such as trastuzumab, pertuzumab, trastuzumab emtansine (T-DM1), and lapatinib [,^4^,^5^]. Given the recent availability of novel and extremely active anti-HER2 therapies, such as trastuzumab deruxtecan (T-DXd) and tucatinib as second or third line of treatment for HER2+ MBC, patients’ outcome is further expected to ameliorate over the next years.

However, not all patients achieve optimal benefit from anti-HER2 therapies, and clinical trials and real-world evidences indicate that despite the major therapeutic advances for patients facing an initial metastatic diagnosis, nearly all will require additional treatments when cancer progresses and will eventually die from their disease [6]. It is therefore critical to understand the unmet medical needs of this patient population in order to better address them in clinical practice.

Cancer registries and real-word databases are useful tools to evaluate the impact of therapeutic changes on patients’ outcomes over long periods of time and to identify special subgroups that may benefit less from current treatments. In the present study, we evaluated how therapeutic approaches to HER2+ MBC changed over time by analyzing first and later lines of treatment and their impact on PFS using a large real-world database. We also aimed to identify subgroups of patients that continue to have poor outcomes, regardless of the most novel treatment received, to provide insights on critical areas to which most of the scientific efforts should be directed.

## 2 Materials and methods

### 2.1 Study design and patient population

The present analysis was conducted within the GIM14/BIOMETA study (ClinicalTrials.gov identifier: NCT02284581), a retrospective/prospective multicenter observational study of the Gruppo Italiano Mammella (GIM) Study Group regarding treatment patterns and outcomes of patients with MBC. For the present analysis, retrospective/prospective clinicopathologic data on patients with HER2+ MBC receiving first and further lines of treatment for metastatic disease between November 2000 and 2020 were retrieved. Patients received treatments and were followed according to routine clinical care in the respective institutions. This study was approved by the institutional review boards of each participating institution and written informed consent was required for patients enrolled in the prospective part of the study, according to Italian law.

### 2.2 Data collection

All the data used for the analysis were derived from the GIM14/BIOMETA electronic database. For each patient, we retrieved information on (neo)adjuvant treatments, distant recurrence, and treatment history for metastatic disease. Tumor response was assessed locally by treating physicians. Hormone receptors (HR) status, Ki67, and HER2 expression were determined locally by the pathologists at participating centers. HR and HER2 status were assigned according to the 2010 and 2013 ASCO/College of American Pathologists guidelines, respectively [7]. From the 2985 patients included in the GIM4/BIOMETA database, a total of 667 women with HER2 positive MBC were identified. Current analyses was performed on 637 patients as 30 patients have been excluded because of lacking information on treatment received.

### 2.3 Study objectives

The primary objective was to describe first and later lines of therapy and their outcomes in patients with HER2+ MBC. First-line PFS was analyzed in the overall population according to the type of anti-HER2 treatment, disease presentation (i.e., de novo vs. recurrent), metastatic site at diagnosis, and year of treatment (2000–2013 vs. 2014–2020). For PFS analyses according to metastatic sites, the specific metastatic site considered per each analyses is considered regardless the co presence of other metastatic sites. The following sites of visceral metastases were considered in the analysis: liver, lung, and metastases in the central nervous system. The following sites of non-visceral metastases were considered in the analysis: bone, tegumental (breast, skin, lymph nodes and soft tissues). Second-line PFS was analyzed in the overall population and according to type of anti-HER2 treatment and year of treatment (2000–2013 vs. 2014–2020).

### 2.4 Statistical analysis

PFS was defined as the time between start of treatment and progression or death, or last assessment for patients alive without progression. Median follow-up (mFU) was calculated according to the reverse Kaplan-Meier technique [8]. PFS curves for the overall population and according to prespecified subgroups were estimated by the Kaplan-Meier method [9] and compared with the log-rank test. Prespecified subgroups were identified according to type of the anti-HER2 therapy received, specific metastatic site at the first diagnoses of MBC and year of treatment. Hazard ratios (HRs) of PFS with 95% confidence intervals (CIs) were calculated in univariable Cox regression models. An interaction test was applied to explore the heterogeneity of the PFS outcome according to each site of metastatic disease in the two periods of treatment start (2000–2013 and 2014–2020). The *P* value for interaction was calculated in a Cox model including the site of metastatic disease, the period of treatment start, and their interaction. A *P* value < 0.05 was considered statistically significant. Considering the descriptive and exploratory intent of the analysis, no adjustment for multiple testing was applied. Statistical analyses were performed with SPSS for Windows, version 27.0.

## 3 Results

### 3.1 Population characteristics

The GIM14/BIOMETA database includes data on 2985 patients with MBC treated between 2000 and 2020. Overall, data from 637 patients with HER2+ MBC treated in the advanced setting between 2000 and 2020 within participating institutions were available for the present study. At the time of diagnosis, 422 (66%) and 215 (34%) patients had early and de novo MBC, respectively. Median age at the start of first-line treatment for MBC was 55 years (range, 45–64). Among patients with early BC (EBC) at diagnosis, data on baseline pathologic stage was available for 363 (86%) patients. In detail, 177 (49%), 137 (37%), 28 (8%), and 21 (6%) were T1, T2, T3, and T4, respectively. Regarding pathologic nodal status, information was available for 369 (87%) patients. In detail, 4 (1%), 125 (34%), 112 (30%), 65 (18%), 63 (17%) were classified as Nx, N0, N1, N2, and N3, respectively. Of the patients with EBC, 257 (61%) received anti-HER2-based therapy (mainly trastuzumab and T-DM1) as adjuvant or neoadjuvant therapy. At the onset of metastatic disease, bone, lymph nodes, and liver were the most common metastatic sites. The main baseline demographic characteristics are summarized in Table 1.

**Table 1:** Descriptive characteristics of patients incuded in the study.

Baseline characteristics	Number (%)
Age (N = 637)	
Median (years); min-max range	55; 45-64
Menopausal state (N = 637)	
Premenopausal	215 (34)
Postmenopausal	381 (60)
Unknown	41 (6)
Metastatic at diagnosis (N = 637)	
Yes	215 (34)
No	422 (66)
Neoadjuvant trastuzumab (N = 422)	
Yes	55 (13)
No	367 (87)
Neoadjuvant pertuzumab and trastuzumab (N = 422)	
Yes	1 (0.24)
No	421 (99.76)
Adjuvant trastuzumab (N = 422)	
Yes	200 (47)
No	222 (53)
Adjuvant T-DM1 (N = 422)	
Yes	1 (1)
No	421 (99)
Site of metastases (N = 601)	
Bone	261 (43)
Lymph nodes	257 (43)
Liver	185 (31)
Lung	155 (26)
Breast	74 (12)
Brain	53 (9)
Skin	51 (8)
Pleura	25 (4)
Soft tissue	12 (2)
Peritoneum	2 (1)
Other	26 (4)

### 3.2 First-line therapy and outcomes according to prespecified subgroups

**Fig. 1:**
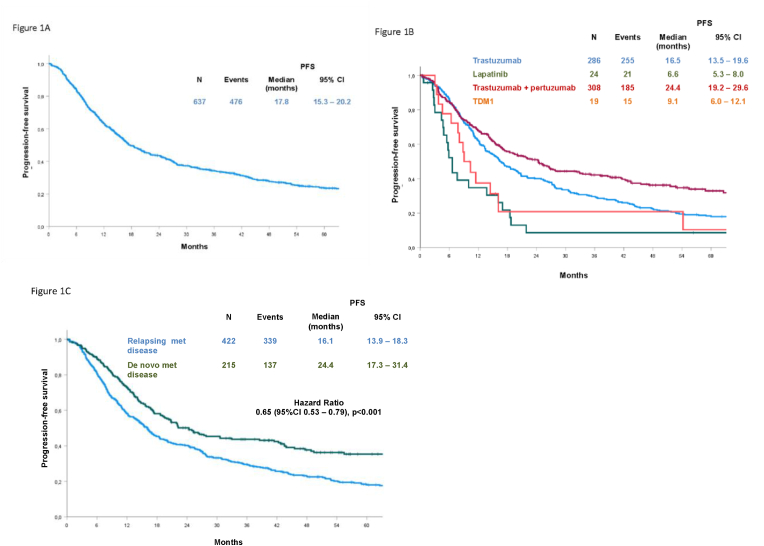
Progression-free survival in patients treated by first-line therapy in the overall study population (A), according to the type of anti-HER2 therapy (B) and metastatic disease presentation (C).

All 637 patients included in the present analysis received anti-HER2 therapy at onset of metastatic disease. Pertuzumab plus trastuzumab and trastuzumab alone were the most commonly prescribed anti-HER2 agents (Table 2). At a mFU of 64.4 months, with 476 events, the overall PFS for patients on first-line treatment was 17.8 months (95% CI, 15.3–20.2) (Fig. 1, A), with significant differences according to treatment type, metastatic status at first diagnosis, metastatic sites, and year of treatment. Regarding the type of anti-HER2 regimen received, the mPFS was 24.4 months (95% CI, 19.12–29.6) with a trastuzumab + pertuzumab-based regimen and 16.5 months (95% CI, 13.5–19.6) with trastuzumab. In the case of first-line T-DM1 or lapatinib-based treatment, the mPFS was 9.1 months (95% CI, 6.0–12.1) and 6.6 months (95% CI, 5.3–8.0), respectively (Fig. 1, B). The differences observed were statistically significant (*P* < 0.001). mPFS was better for patients with de novo metastatic disease (24.4 months; 95% CI, 17.3–31.4) compared with those who relapsed (16.1 months; 95% CI, 13.9–18.3) (Fig. 1, C) (HR, 0.65; 95% CI, 0.53–0.79, *P* < 0.001). Overall, mPFS was not significantly affected by the presence of non visceral (breast, lymph nodes, skin, soft tissues), bone, lung, or liver metastatic involvement. PFS was 18.7 months (95% CI, 15.1–22.2) versus 17.1 months (95% CI, 13.1–21.0) (*P* = 0.45) in the presence versus absence of non visceral metastases; 19.4 months (95% CI, 14.1–24.6) versus 17.4 months (95% CI, 14.9–19.9) (*P* = 0.65) in the presence versus absence of bone metastases; 16.4 months (95% CI, 12.3–20.4) versus 18.7 months (95% CI, 14.8–22.4) (*P* = 0.83) in patients with or without lung metastases; 16.1 months (95% CI, 12.4–19.8) versus 19.1 months (95% CI, 15.2–23.0) (*P* = 0.09) in the presence versus absence of liver metastases. In contrast, the presence of central nervous system (CNS) metastases was associated with a significantly worse PFS. The mPFS was 8.9 months (95% CI, 6.4–11.5) versus 19.5 months (95% CI, 16.2–22.8) (HR, 1.75; 95% CI, 1.29–2.38, *P* < 0.001) in patients with versus without CNS localizations.

**Table 2:** Descriptive characteristics of patients’ treatment.

Treatment characteristics	Number (%) 637 (100)
Type of anti-HER2 for 1st line treatment	
Pertuzumab + trastuzumab ± other	308 (48)
Trastuzumab ± other	286 (45)
Lapatinib ± other	24 (4)
T-DM1 ± other	19 (3)
Type of treatment for 1st line treatment	
Trastuzumab + monochemotherapy (vinorelbine or taxanes)	187 (29)
Trastuzumab + polychemotherapy including anthracyclines	36 (6)
Trastuzumab + polychemotherapy without anthracyclines	14 (2)
Trastuzumab + hormonal treatment	49 (8)
Pertuzumab + trastuzumab + monochemotherapy (taxanes)	251 (39)
Pertuzumab + trastuzumab + anthracycline-based polychemotherapy	8 (1)
Pertuzumab + trastuzumab + polychemotherapy without anthracyclines	11 (2)
Pertuzumab + trastuzumab + hormonal treatment	38 (6)
Lapatinib + hormonal treatment	24 (4)
T-DM1	19 (3)
Type of anti-HER2 for 2nd line treatment	
Trastuzumab ± hormonal treatment or chemotherapy	95 (27)
Lapatinib + capecitabine	83 (23)
Pertuzumab + trastuzumab ± hormonal treatment or chemotherapy	21 (6)
T-DM1	156 (44)
Other	1 (0.3)
Any anti-HER2 (total)	356 (56)
Type of anti-HER2 for 3rd line treatment	
Trastuzumab ± hormonal treatment or chemotherapy	88 (40)
Lapatinib + capecitabine	80 (36)
Pertuzumab + trastuzumab ± hormonal treatment or chemotherapy	3 (1)
T-DM1	48 (22)
Any anti-HER2 (total)	219 (34)
Type of anti-HER2 for 4th line treatment	
Trastuzumab ± hormonal treatment or chemotherapy	81 (63)
Lapatinib + capecitabine	25 (20)
T-DM1	22 (17)
Any anti-HER2 (total)	128 (20)
Type of anti-HER2 for 5th line treatment	
Trastuzumab ± hormonal treatment or chemotherapy	38 (79)
Lapatinib + capecitabine	7 (15)
T-DM1	3 (6)
Any anti-HER2 (total)	48 (8)

### 3.3 First-line therapy PFS according to the year of treatment

**Fig. 2:**
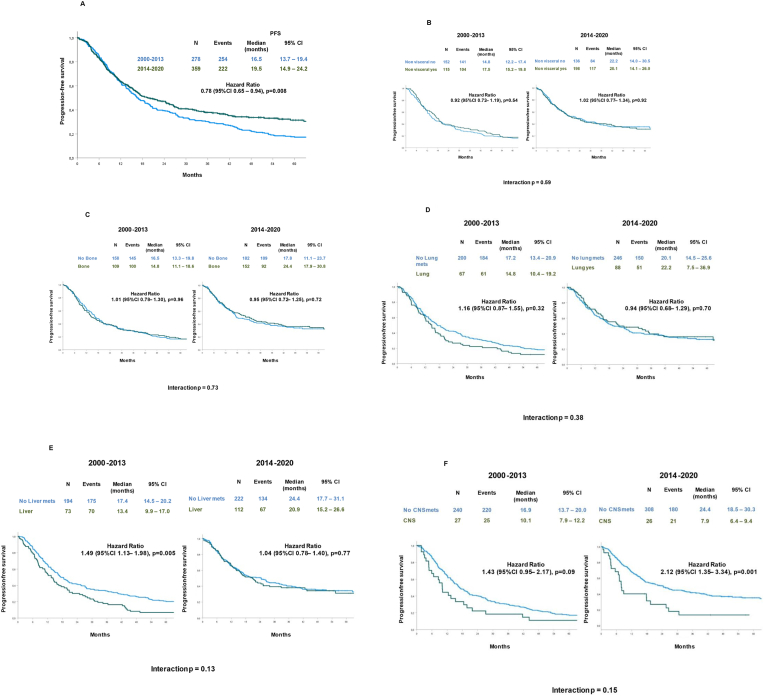
Progression-free survival according to year of diagnosis for metastatic disease in the overall population (A), in patients with or without non visceral (B), bone (C), lung (D), liver (E), and CNS (F) metastases.

In Italy, pertuzumab became available in most Italian hospitals after 2013. In our database, 237 of 278 (85.3%) patients starting first-line therapy between 2000 and 2013 received trastuzumab alone and 287 of 359 (79.9%) patients starting first-line therapy between 2014 and 2020 received trastuzumab plus pertuzumab as anti-HER2 therapy. Median PFS was 16.5 months (95% CI, 13.7–19.4) for patients treated in 2000–2013 and 19.5 months (95% CI, 14.9–24.2) for patients treated in 2014–2020 (HR, 0.78, 95% CI, 0.65–0.94, *P* = 0.008) (Fig. 2, A).

No relevant interaction was evident in terms of PFS between the year treatment started and the presence of non visceral (*P* = 0.59), bone (*P* = 0.73), or lung (*P* = 0.38) metastases (Fig. 2B–D). Despite the absence of significant interaction (*P* = 0.13), when comparing presence versus absence of liver metastases, a significant difference in PFS was observed according to the year treatment started. A significantly worse PFS was observed for patients with versus without liver metastases (13.4 months, 95% CI, 9.9–17.0 vs. 17.4 months, 95% CI, 14.5–20.2, respectively) in the population treated in 2000–2013 (HR, 1.49; 95% CI, 1.13–1.98, *P* = 0.005). Conversely, no difference was observed in the population treated in 2014–2020 according to the presence/absence of liver disease (*P* = 0.77) (Fig. 2, E). In contrast to liver metastasis, the presence of CNS metastasis was associated with a non-significant trend toward a worse prognosis in the older cohort; the mPFS of patients with versus without CNS metastases was 10.1 months (95% CI, 7.9–12.2) versus 16.9 months (95% CI, 13.7–20.0) (*P* = 0.09). Conversely, a statistically significant difference was observed in the 2014–2020 cohort according to CNS localization; the mPFS was 7.9 months (95% CI, 6.4–9.4) in the case of brain involvement versus 24.4 months (95% CI, 18.5–30.3) in the absence of CNS metastases (HR, 2.12; 95% CI, 1.35–3.34; *P* = 0.001). However, there was no significant interaction between involvement of CNS metastasis and the year treatment started (*P* = 0.15) (Fig. 2, F).

### 3.4 Second-line therapies and outcomes

Overall, 356 (56%) patients received a second-line treatment with an anti-HER2 agent (Table 2).

**Fig. 3:**
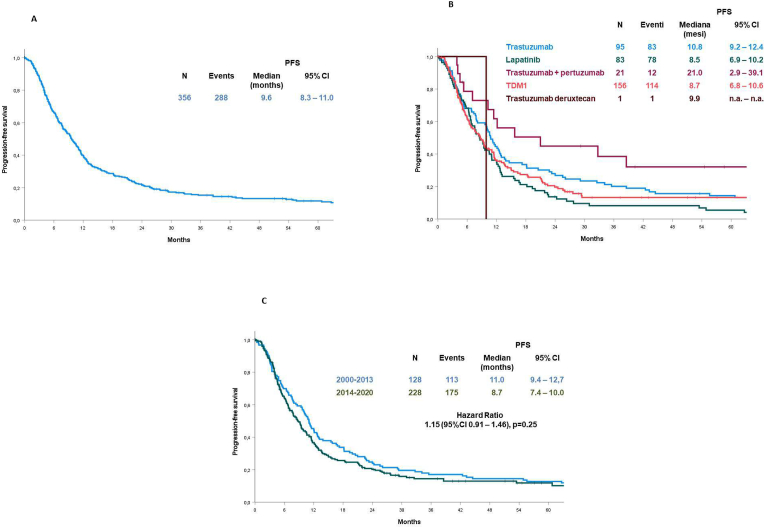
Progression-free survival in 356 patients treated by second-line therapy in the overall study population (A), according to the type of anti-HER2 therapy (B) and the period of treatment (C).

Overall, the mPFS for patients treated with a second-line regimen was 9.6 months (95% CI, 8.3–11.0) (Fig. 3, A). According to the treatment administered, the mPFS was 8.7 months (95% CI, 6.8–10.6) with T-DM1, 10.8 months (95% CI, 9.2–12.4) with trastuzumab, 8.5 months (95% CI, 6.9–10.2) with lapatinib, 21.0 months (95% CI, 2.9–39.1) with trastuzumab plus pertuzumab and 9.9 months with other therapy (Fig. 3, B). The difference was statistically significant (*P* < 0.001). The mPFS was 11.0 months (95% CI, 9.4–12.7) for patients starting treatment up to 2013 versus 8.7 months (95% CI, 7.4–10.0) for patients starting treatment from 2014 (HR, 1.15; 95% CI, 0.91–1.46), but the observed difference was not statistically significant (*P* = 0.25) (Fig. 3, C).

### 3.5 Third and later lines

**Fig. 4:**
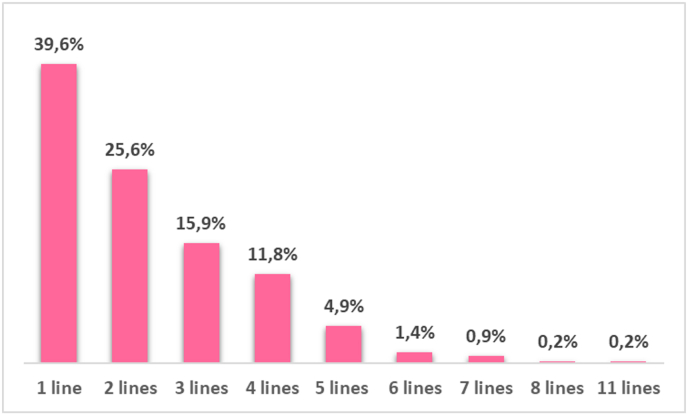
The total number of lines of anti-HER2 treatment for metastatic disease in the overall population (*N* = 637) with a median follow-up of 64.4 months.

**Fig. 5:**
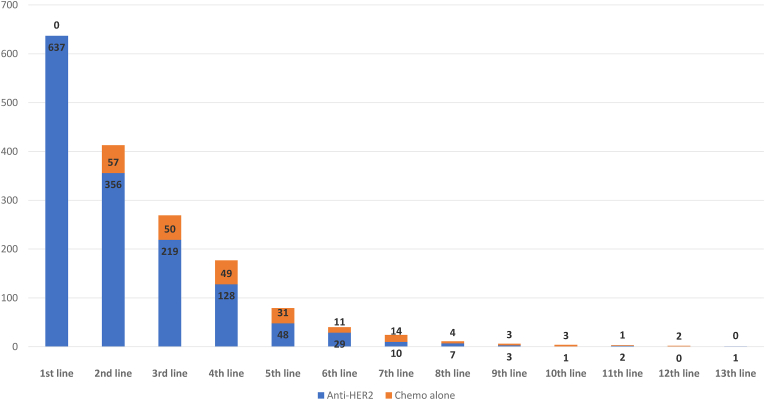
Number of patients receiving each line of treatment with and without anti HER2 therapy.

In our dataset, 219 (34%), 128 (20%), and 48 (8%) patients received a third, fourth and fifth anti-HER2-based treatment, respectively (Table 2, Fig. 4). Among third-line therapies, 88 (40%) patients received trastuzumab with chemotherapy, while 80 (36%) patients received lapatinib with chemotherapy. Forty-eight patients (22%) were treated with T-DM1 and 3 (1%) with trastuzumab plus pertuzumab and chemotherapy. Among fourth-line therapies, trastuzumab with different chemotherapy regimens was the most prescribed anti-HER2-bsed regimen, with 81 (63%) patients receiving such regimens. Lapatinib plus capecitabine and T-DM1 were delivered in 25 (19%) and 22 (17%) patients, respectively. Regarding fifth-line therapy: 38 (79%), 7 (15%) and 3 (6%) patients received trastuzumab, lapatinib with chemotherapy and T-DM1, respectively. According to the inclusion criteria of our analysis, as shown in Fig. 5, no patient received chemotherapy without anti-HER2 therapy as first line of therapy. However, a minority of patients received chemotherapy without anti-HER2 therapies in second and later lines, probably due to toxicity from anti HER2 therapies or, for patients treated in the earlier years of our period of observation, for the uncertainty about the benefit of continuing anti her2 therapies beyond progression to first line trastuzumab. Interestingly drop-off rate from one line to another among patient receiving chemo without anti-HER2 therapy was less pronounced for second third and fourth line compared to antiher2 therapiesGiven the small number of patients per each line, no further PFS analyses was performed in these subgroups.

## 4 Discussion

This observational, multicenter, retrospective/prospective study reports real-life data on treatment patterns and outcomes in terms of PFS for 637 patients with HER2+ MBC enrolled in the Italian BIOMETA study. First-, second-, third-, fourth-, and fifth-line treatments were analyzed for the whole population. Based on the timespan for which data were available, the most relevant therapeutic improvements after the advent of trastuzumab were the introduction of pertuzumab and T-DM1, therefore we split the population according to their introduction into clinical practice in Italy. Patients were subdivided into a group treated for the first time for their MBC between 2000 and 2013 (before pertuzumab and T-DM1) and a group treated for the first time from 2014 to 2020 (after availability of pertuzumab and T-DM1).

The overall PFS after first-line therapy in our study population was 17.8 months (95% CI, 15.3–20.2), which is in line with those reported in literature, considering that half of the patients did not receive pertuzumab [1]. Not surprisingly, PFS was significantly prolonged by pertuzumab-based therapy, reaching 24.4 months in patients undergoing anti-HER2 double blockade. Our results are comparable with those observed in other real-world studies [[10], [11], [12]], but slightly different from those reported in the CLEOPATRA randomized registration trial and the phase IIIb Peruse trial, where the mPFS in the pertuzumab arm was 18.7 months and 20.7 months, respectively (8, Miles et al. Annals Oncol 2021 PMID 34224826). Different timing of tumor response assessment may explain the differences observed between real-word and registration studies, as well as the proportion of relapsing versus de novo disease, the distribution of metastatic sites, and early-stage systemic treatments. In the present study, the mPFS of de novo versus relapsing patients was 24.4 versus 16.1 months, confirming similar results from the CLEOPATRA trial, where a higher benefit from pertuzumab was reported in patients with de novo metastatic disease [13]. Analyzing outcome trends according to the year of diagnosis, PFS for patients diagnosed in 2000–2013 versus 2014–2020 was significantly shorter, further confirming the positive impact of pertuzumab on MBC outcome.

According to our previous data, the most commonly reported single sites for distant metastases at diagnosis for MBC are bone and lymph nodes, followed by lung, liver, and brain [14]. The presence/absence of bone, lung, or liver metastases did not have an impact on PFS in our population. However, when PFS was analyzed according to metastatic site and year of treatment, we found that for patients treated up to 2013 (ie, treated with trastuzumab alone), the presence of liver metastatic disease was significantly associated with worse PFS. The introduction of pertuzumab in this subgroup dramatically improved outcomes because differences in PFS based on liver metastatic status were no longer observed after 2014. This evidence seems to confirm the subgroup analyses in the CLEOPATRA trial, which showed that patients with visceral disease benefited most from addition of pertuzumab [15].

In our study, the incidence of brain metastasis was 9%. The prevalence of brain metastasis in patients with HER2+ MBC vary greatly among individual studies [16,17]. A meta-analysis of results from 25 studies indicated a cumulative incidence of 31% (13% per patient-year) [18]. Patients with brain metastasis had a significantly shorter PFS than those without in our cohort. The poorer outcome in this subset did not improve with the addition of pertuzumab as shown by the PFS of patients treated after 2014, who received pertuzumab-based therapy in 80% of cases. The lack of outcome improvement was observed despite better local therapies (i.e., neurosurgery and stereotactic radiotherapy) delivered in later years, meaning that, in this subgroup of patients, it is critical to further improve systemic therapies to significantly improve outcomes. Novel anti-HER2 agents such as tucatinib and trastuzumab deruxtecan (T-DXd) have demonstrated clear improvements in patients with brain metastasis [[19], [20], [21], [22], [23]] with intracranial response rate of 43% and 73.3% in the HER2CLIMB [21] and TUXEDO-1 [22] trials, respectively. Therefore, a more rapid and broader delivery of such important innovative therapeutic agents to clinical practice should be strongly advocated.

In this patient cohort, second-line therapy was given to 56% patients, achieving a mPFS of 9.6 months. Almost half the population received T-DM1. The efficacy observed in terms of mPFS was similar to that observed in the EMILIA T-DM1 2nd-line pivotal trial but, surprisingly, no difference was observed in the two different time cohorts. An unexpected numerically better outcome was observed for the 2000–2013 cohort. It is possible that this is due to the reduced use of trastuzumab in early settings for this subgroup, which might have somewhat improved the efficacy of anti-HER2 agents in the advanced disease. The recent approval for Trastuzumab Deruxtecan for second and later lines of treatment for HER2+ MBC is expected to importantly improve patients outcome in this setting and further studies are warranted to evaluate the impact of this new treatment on large real world patient populations. In this perspective, the impact of the recent addition of pertuzumab and T-DM1 to the therapeutic armamentarium of the early-stage disease have to be carefully evaluated in the next future [24,25], since less relapses, but in more highly anti-HER2 pre-treated patients will likely be observed.

Results from clinical trials and real-world studies indicate that despite major therapeutic advances, nearly all patients will require additional treatment when their cancer eventually progresses. Attrition of patients with HER2+ MBC across lines of therapy has been observed in multiple studies; 18%–45% of patients who experience disease progression during first-line treatment, drop out and do not receive further treatment. Another 10%–55% attrition between second- and third-line therapy has been also observed [26,27]. Consistent with these data, in our study, only 56% of patients received at least a second line of anti-HER2 therapies, and only 20% of patients received more than 3 lines of anti-HER2-based regimens. Reasons why patients dropped out from one line of treatment to the next were not described in the database. However, age, performance status, and treatment tolerability are currently considered the key factors in the decision to prescribe further anti-tumor treatment versus supportive care among physicians. About 25% of patients being treated for HER2+ MBC die within the first 20 months of treatment [28]. Thus, it may be reasonable to give the most effective regimens in the earliest possible line of treatment because of the risk of not getting to a subsequent line due to rapid disease progression or development of complications.

The main limitations of the present study are inherent to the general limitations of real-world observational studies, and include lack of randomization, lack of uniform timing or type of clinical assessments, and challenges with missing data. Monitoring toxicity was not a prespecified endpoint in the GIM14/BIOMETA study protocol. Given the very well characterized and known toxicity profile of the anti HER2 drugs included in our analysis safety data was not further proactively collected and presented in this study.We could not assess survival rate for patients included in the study because data were not readily available at the time of the analysis. Further data retrieval for OS evaluation are currently ongoing. Despite these limitations, our study includes a large multi-institutional treated population of patients with HER2+ MBC and provides a clear insight into prescriptive patterns and relative outcomes over time. The heterogeneity of the patient characteristics included in this analysis may further strengthen our findings, making them applicable to a larger population of patients, and closer to routine clinical practice.

## 5 Conclusions

Clinical practice and treatment patterns have changed dramatically over time in the setting of HER2+ MBC. The improvements may be mostly due to the higher efficacy of more advanced treatments such as pertuzumab. However, despite the huge advantages observed, there are still patients who do not experience the same degree of improvement such as those with brain metastases. Emerging therapies such as T-DXd and tucatinib are extremely promising and quicker implementation in current clinical practice should be urgently pursued. Our data also support the use of the best available drugs as early as possible, because many patients drop out from oncologist care following the first/second line of therapy.

## Funding

BIOMETA/GIM14 study has been partially supported by Roche.

Medical writing support for the preparation of this article was provided by Michela Roberto, on behalf of Edra S.p.A., and with an unrestricted grant by 10.13039/100004325AstraZeneca.

## Role of the funder

The funder did not play a role in the design of the study; the collection, analysis, and interpretation of the data; the writing of the manuscript; and the decision to submit the manuscript for publication.

## Informed consent

This study is non interventional and was approved by the institutional review boards of each participating institution and written informed consent was required for patients enrolled in the prospective part of the study, according to Italian law.

## Author contributions

All Authors have made a substantial contribution to the concept or design of the article; or the acquisition, analysis, or interpretation of data for the article; AND.

Drafted the article and revised it critically for important intellectual content AND.

Approved the version to be published; AND.

Agreed to be accountable for all aspects of the work in ensuring that questions related to the accuracy or integrity of any part of the work are appropriately investigated and resolved.

## Data availability statement

The anonymized database is available from the corresponding author upon reasonable request.

## Declaration of competing interest

G.A. reports support for the present manuscript from Astra Zeneca and Istitutional Grant from Astra Zeneca; consulting fees from Astra Zeneca, Daiichi-Sankyo, Novartis, Roche, Pfizer, Eli Lilly, Gilead, Seagen; personal fees for lectures, presentations, speakers bureaus, manuscript writing or educational events from Astra Zeneca, Daichii Sankyo, Eisai, Eli Lilly, Exact Sciences, Gilead, Novartis, Roche, Seagen, Viatris; support for attending meetings and/or travel from Daichii - Sankyo, Roche, Novartis; participation on a Data Safety Monitoring Board or Advisory Board for Astra Zeneca, Daichii - Sankyo, Eisai, Eli Lilly, Gilead, Novartis, Roche, Seagen.

C.B. reports personal fees for lectures, presentations, speakers bureaus, manuscript writing or educational events from Roche, Eli Lilly, Novartis, Pfizer; personal fees for support for attending meetings and/or travel from Roche, Novartis; personal fees for participation on a Data Safety Monitoring Board or Advisory Board from Roche, Novartis.

C.D.A. reports personal fees from AstraZeneca, Lilly, GSK, Novartis, Seagen, and Pfizer, Advisory Board for Roche, AstraZeneca, Lilly, GSK, Novartis, Seagen, and Pfizer, support for attending meetings and/or travel AstraZeneca, Lilly, GSK, Novartis, Celgene, and Pfizer, grants (to the Institution) from Novartis.

M.D.L. reports payment or honoraria for lectures, presentations, speakers bureaus, manuscript writing or educational events from Eli Lilly, Menarini; support for attending meetings and/or travel from Gilead, Roche, Astra Zeneca, Novartis, Roche, Pfizer, Gilead, Seagen, Daiichi-Sankyo, Pfizer, Eli Lilly, Genetic, Pierre Fabre, GSK, Exact Sciences, Tomalab, Eisai, MSD and participation on a Data Safety Monitoring Board or Advisory Board for Seagen, Eli Lilly, Novartis, Pierre Fabre, Sanofi Genzyme, GSK, Gilead, Astra-Zeneca, Daiichi-Sankyo, MSD; Roche, Eisai, Exact Scences.

L.D.M. reports grants to the Institution for patient enrollment in studies from Eli Lilly, Novartis, Roche, Daiichi - Sankyo, Seagen; consulting fees from Ely Lilli, Roche, Novartis, Pfizer, Astra Zeneca, MSD, Seagen, Gilead, Pierre Fabre, Eisai, Exact sciences, Ipsen, Agendia, GSK, Daiichi – Sankyo; support for attending meetings and/or travel from Roche, Pfizer, Eisai, Daiichi - Sankyo; participation on a Data Safety Monitoring Board or Advisory Board for Novartis, Roche, Eli Lilly, Pfizer, Daiichi - Sankyo, Exact sciences, Gilead, Pierre Fabre, Eisai, Astra Zeneca, Agendia, GSK.

M.D.M reports personal fees for Advisory boards or consultant role: Astra Zeneca, Novartis, Roche, Pfizer, Eisai, MSD, Janssen, Boehringer Ingelheim, Takeda, Amgen, Merck, Servier and research grant to the Institution: Tesaro, GlaxoSmithKline.

A.F. reports payment or honoraria for lectures, presentations, speakers bureaus, manuscript writing or educational events from Roche, Astra Zeneca, Eli Lilly, Pfizer, Novartis, Gilead; support for attending meetings and/or travel for Astra Zeneca, Roche, Gilead, Eli Lilly; participation on a Data Safety Monitoring Board or Advisory Board for Roche, Novartis, Lilly, Pfizer, MSD, Dompè, Pierre Fabre, Eisai, Sophos, Epionpharma, Gilead, Seagen, Astra Zeneca, Exact Science.

M.G. reports consulting fees from Lilly, Celgene, Novartis, Pfizer, Astra Zeneca, Daiichi-Sankyo, MSD, Gilead, payment or honoraria for lectures, presentations, speakers bureaus, manuscript writing or educational events for Lilly, Celgene, Novartis, Pfizer, Istituto Gentili, Eisai Europe Ltd, Roche Astra Zeneca, Daiichi - Sankyo, MSD, Gilead, support for attending meetings and/or travel from Novartis, Pfizer, Roche.

L.M. reports personal fees from MSD for lectures, presentations, speakers’ bureaus, manuscript writing or educational events and support for attending meetings and/or travel from Janssen.

F.P. reports consulting fees from Astra Zeneca, Daichii - Sankyo, Novartis, Roche; payment or honoraria for lectures, presentations, speakers bureaus, manuscript writing or educational events from Astra Zeneca, Daichii – Sankyo, Eisai, Eli Lilly, Exact Sciences, Gilead, Novartis, Roche, Seagen, Viatris; support for attending meetings and/or travel from Daichii - Sankyo, Roche; participation on a Data Safety Monitoring Board or Advisory Board for Astrazeneca, Daichii – Sankyo, Eisai, Eli Lilly, Gilead, Novartis, Roche, Seagen, Viatris.

F.R reports payment or honoraria for lectures, presentations, speakers bureaus, manuscript writing or educational events from Roche, Astra Zeneca, Lilly, Pfizer, Novartis, Gilead; support for attending meetings and/or travel from Astra Zeneca, Roche, Gilead, Lilly; participation on a Data Safety Monitoring Board or Advisory Board for Roche, Novartis, Eli Lilly, Pfizer, MSD, Dompè, Pierre Fabre, Eisai, Sophos, Epionpharma, Gilead, Seagen, Astra Zeneca, Exact Science.

F.S. reports honoraria for presentations and educational materials from Novartis.

Authors not mentioned declare that the research was conducted in the absence of any commercial or financial relationships that could be construed as a potential conflict of interest.
